# Prevalence, spatial distribution, and determinants of anemia among under-five children in the Democratic Republic of Congo: evidence from the 2023–24 DHS

**DOI:** 10.1186/s12942-026-00484-4

**Published:** 2026-07-29

**Authors:** Thomas kidanemariam Yewodiaw, Helen Lamesgin Endalew, Mihret Getnet, Amare Belete Getahun, Tiget Ayelgn Mengstie, Engidaw Fentahun Enyew, Hiwot Tezera Endale, Tseganesh Asefa, Mequanent Dessie Bitewa

**Affiliations:** 1 International Medical Corps, Amhara Region Emergency Operation Center, Gondar Field Office, Gondar, Ethiopia; 2 Department of Surgical Nursing, School of Nursing, College of Medicine and Health Sciences, University of Gondar, Gondar, Ethiopia; 3 Department of Human Physiology, School of Medicine, College of Medicine and Health Sciences, University of Gondar, Gondar, Ethiopia; 4 Department of Anesthesia, School of Medicine, College of Medicine and Health Sciences, University of Gondar, Gondar, Ethiopia; 5 Department of Medical Biochemistry, School of Medicine, College of Medicine and Health Sciences, University of Gondar, Gondar, Ethiopia; 6 Department of Human Anatomy, School of Medicine, College of Medicine and Health Sciences, University of Gondar, Gondar, Ethiopia; 7 Department of Reproductive Health, Institute of Public Health, College of Medicine and Health Science, University of Gondar, Gondar, Ethiopia; 8 Armauer Hansen Research Institute, Addis Ababa, Ethiopia; 9 Department of Epidemiology and Biostatistics, Institute of public Health, College of Medicine and Health Sciences, University of Gondar, Gondar, Ethiopia; 10 Department of Public Health, College of Health Sciences, Debre Markos University, Debre Markos, Ethiopia

**Keywords:** Anemia, Under-five children, Democratic Republic of Congo, DHS, Multilevel analysis, Spatial distribution, Hotspot analysis

## Abstract

**Background:**

Anemia among children under five remains a major public health problem in the Democratic Republic of Congo (DRC), affecting growth, cognitive development, and overall child health. We estimated the prevalence, spatial distribution, and determinants of anemia and identified high-risk geographic areas using multilevel and spatial analyses.

**Methods:**

We analyzed data from 11,393 children aged 6–59 months from the 2023–24 Democratic Republic of Congo Demographic and Health Survey (DHS). Weighted prevalence estimates were calculated, and multilevel mixed-effects logistic regression was used to assess individual- and community-level determinants of anemia. We applied Moran’s I, hotspot detection, Kriging interpolation, and SaTScan analyses to identify high-risk areas. Model performance was evaluated using AIC, BIC, and log-likelihood ratio tests to ensure the results were reliable.

**Results:**

Overall, 51.7% of children (95% CI: 49.5%–53.9%) were anemic, most cases were mild (25.6%) or moderate (25.0%), while severe anemia was rare (1.1%). Prevalence was highest among infants 0–5 months (56.2%) and boys (52.5%) than girls (50.8%). Key risk factors for anemia included wasting (AOR = 1.29), malaria infection (AOR = 1.21), high community malaria risk (AOR = 1.42), and low altitude (AOR = 1.39). Children were less likely to be anemic if their mothers were overweight (AOR = 0.66), if they came from wealthier households (AOR = 0.77), or if they lived in moderately sized families (AOR = 0.84). Spatial analysis revealed high-prevalence areas in Haut-Lomami, Maniema, and Tshuapa, whereas lower prevalence was seen in Haut-Uele and Nord Ubangi.

**Conclusion:**

Anemia among under-five children in the DRC is geographically clustered and influenced by both individual- and community-level factors. Targeted and integrated interventions focusing on malaria prevention, nutrition, and maternal health are needed in high-burden areas.

## Introduction

 Anemia among children under five remains a major public health concern, particularly in sub-Saharan Africa. Globally, nearly 40% of children aged 6–59 months are affected, with anemia contributing to impaired cognitive development, stunted growth, weakened immunity, and increased risks of morbidity and mortality [1, 2]. In sub-Saharan Africa, childhood anemia is driven by nutritional deficiencies, recurrent infections such as malaria and intestinal parasites, maternal anemia, and limited access to healthcare services [3]. Because anemia is multifactorial, its burden is influenced by a combination of individual-, household-, and community-level factors that vary across settings [3–13]. In the Democratic Republic of Congo (DRC), childhood anemia remains highly prevalent and geographically heterogeneous. Previous Demographic and Health Surveys (DHS), including the 2013–14 survey, reported a substantial burden of anemia among under-five children, with marked regional variation across provinces [3]. Local studies from areas such as South Kivu documented prevalence rates as high as 83% among children aged 6–59 months, including 12% with severe anemia [14]. These disparities are likely related to differences in malaria transmission, nutritional conditions, socioeconomic status, and healthcare access across communities [15–17].

Understanding the spatial distribution of anemia is important for identifying high-risk areas and guiding targeted public health interventions. Spatial analytical approaches, including Moran’s I, hotspot analysis, Kriging interpolation, and SaTScan, can help detect geographic clustering and reveal contextual risk patterns that may not be captured through conventional analyses alone [9–11, 18–23]. In addition, multilevel analytical approaches are essential for simultaneously assessing individual-, household-, and community-level determinants of anemia within hierarchical DHS data structures [4, 7, 9, 13, 24, 25]. Despite increasing attention to childhood anemia in sub-Saharan Africa, evidence from the Democratic Republic of Congo remains limited regarding the combined influence of individual, household, community, and geographic factors on childhood anemia. Previous DHS-based studies have mainly focused on national prevalence estimates or conventional regression approaches, providing limited understanding of spatial heterogeneity and localized risk patterns.

This study advances existing evidence by integrating geospatial analysis with multilevel modeling using nationally representative 2023–24 DHS data. The approach allows identification of geographic clusters of childhood anemia and quantifies how individual-level, household-level, community-level, and environmental factors contribute to anemia risk. The findings provide actionable evidence for geographically targeted anemia prevention strategies in high-risk areas of the Democratic Republic of Congo.

## Methods

### Data source and study population

We conducted a cross-sectional study using data from the 2023–24 Democratic Republic of Congo Demographic and Health Survey (DHS), a nationally representative household survey. The DHS employed a stratified two-stage cluster sampling design and collected detailed information on population, maternal and child health, nutrition, mortality, and disease indicators. Our analysis focused on children aged 6–59 months, for whom hemoglobin levels were measured using HemoCue^®^ point-of-care devices according to WHO protocols [26]. Hemoglobin values were adjusted for altitude following WHO guidelines [27]. Sampling weights were adjusted for multilevel modeling because DHS weights are designed for single-level survey estimation. The individual sampling weights were normalized within clusters by dividing each weight by the mean weight of the corresponding cluster, allowing appropriate estimation of fixed and random effects in multilevel models. Ethical approval for the DHS was obtained from the DHS Program and the relevant national review boards in the DRC, and all data were anonymized prior to release [28, 29].

## Inclusion and exclusions criteria

**Fig. 1:**
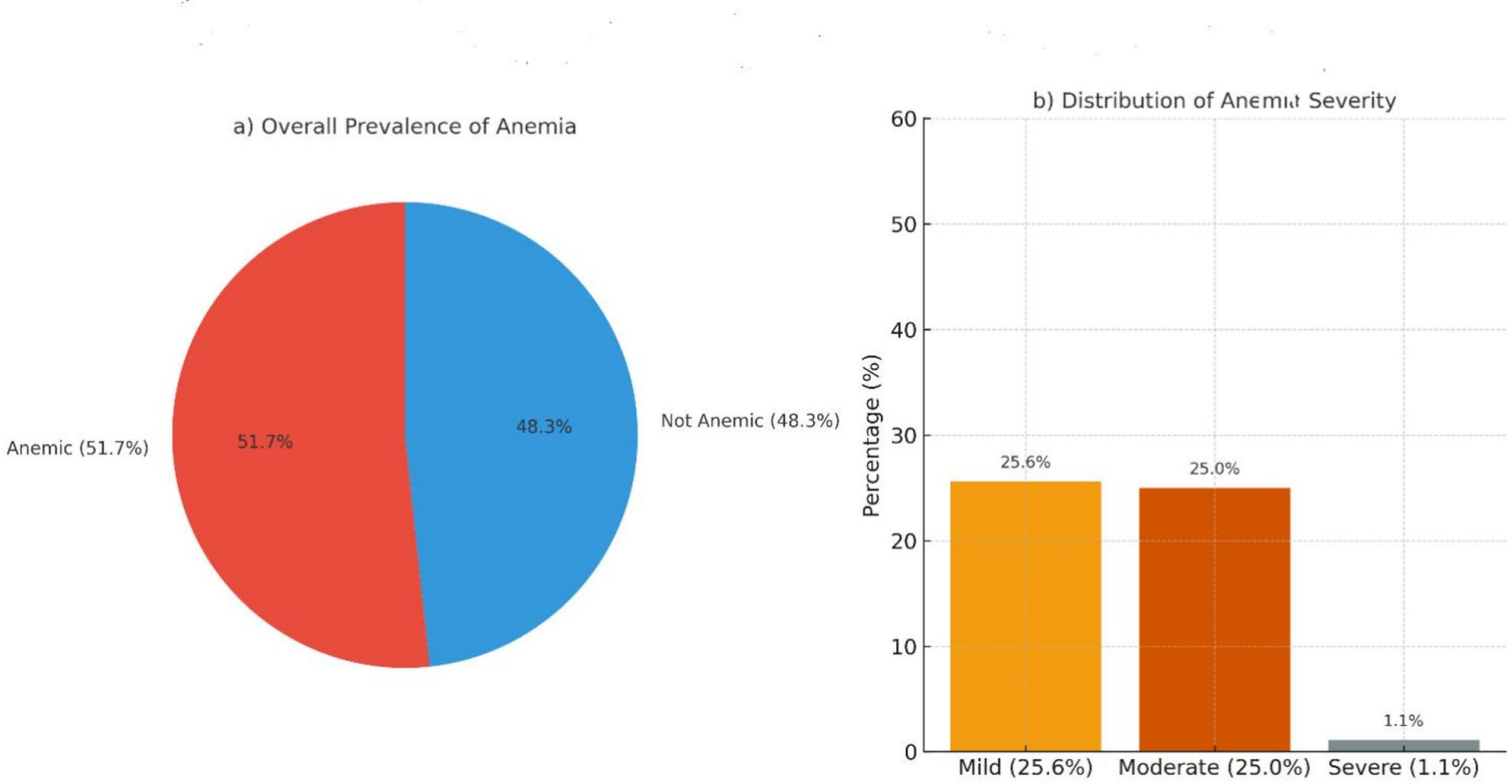
Prevalence and Severity of Anemia Among Under-Five Children in Congo, DHS 2023–24 (Population *N* = 11,393)

Children aged 6–59 months with valid hemoglobin measurements and valid cluster geolocation data were included in the analysis. Children with missing or implausible hemoglobin values or invalid cluster coordinates were excluded. A flow diagram summarizing the inclusion and exclusion process is presented in Fig. 1.

### Outcome Variable

The outcome was anemia, defined using WHO hemoglobin cutoffs for children aged 6–59 months (< 11.0 g/dL) after altitude adjustment. Anemia severity was categorized as mild, moderate, or severe based on WHO thresholds [30, 31].

### Explanatory Variables

We selected anemia determinants based on prior studies and DHS data availability, grouping them into individual, household, and community levels. Individual factors included the child’s age, sex, birth order, nutritional status (stunting, wasting, underweight), and recent illnesses such as fever, malaria infection. Household factors comprised maternal age, maternal education, household wealth, vitamin A supplementation, and deworming. Community-level factors included urban or rural residence, community maternal education, community poverty, community media exposure, and environmental exposures like malaria risk area [4, 7, 15–18, 20, 21, 24, 25, 28–65]. All variables were harmonized and checked for missing data, and DHS sample weights were applied to account for the complex survey design and unequal selection probabilities.

### Operational Definitions

**Anemia** was defined as hemoglobin concentration < 11.0 g/dL for children aged 6–59 months after adjustment for altitude, according to World Health Organization (WHO) guidelines. It was further classified into mild (10.0–10.9 g/dL), moderate (7.0–9.9 g/dL), and severe (< 7.0 g/dL) anemia [66].

**Stunting** was defined as height-for-age Z-score (HAZ) below − 2 standard deviations (SD) from the WHO Child Growth Standards median [67].

**Wasting** was defined as weight-for-height Z-score (WHZ) below − 2 SD, and underweight was defined as weight-for-age Z-score (WAZ) below − 2 SD [67].

**Fever** was defined as a caregiver-reported history of fever in the two weeks preceding the survey [68].

**Malaria infection** was defined based on DHS biomarker testing or reported diagnosis where available [68].

**Household wealth index** was generated by DHS using principal component analysis (PCA) and categorized into quintiles: poorest, poorer, middle, richer, and richest [69, 70].

**Maternal education** was categorized as no formal education, primary education, and secondary or higher education [68].

**Place of residence** was classified as urban or rural based on DHS sampling strata [68] .

**Community-level variables** (e.g., community poverty, maternal education, and media exposure) were derived by aggregating individual-level characteristics at the primary sampling unit (cluster) level and categorized based on national medians [71].

**Malaria endemicity** area was defined using spatial malaria risk classification from external malaria prevalence layers integrated with DHS cluster locations [68].

### Sampling determination and procedure

This study draws on data from the 2023–2024 Democratic Republic of Congo (DRC) Demographic and Health Survey (DHS), which uses a two-stage stratified cluster sampling design. In the first stage, enumeration areas (EAs) were selected with probability proportional to size, stratified by province and urban–rural residence. In the second stage, households were systematically sampled within each EA [42, 43]. Our analysis focused on under five children with valid and reliable hemoglobin (Hb) measurements. DHS presented sampling weights were implemented to adjust for stratification, clustering, and non-response, ensuring nationally representative estimates [42].

### Spatial data preparation

DHS cluster coordinates were randomly displaced to preserve participant confidentiality. Clusters without valid geographic coordinates were excluded. Spatial datasets, including administrative boundaries, malaria prevalence, and relevant environmental risk layers, were prepared to facilitate linkage between anemia outcomes and geographic and ecological factors. It should be acknowledged that DHS spatial displacement (up to 2 km in urban areas and 5–10 km in rural areas) may introduce positional uncertainty, potentially affecting hotspot detection and spatial interpolation accuracy.

### Descriptive and spatial analysis

Descriptive analyses were conducted to estimate the weighted prevalence of anemia, overall, by severity, and across key demographic and clinical characteristics. Survey weights were applied to ensure national representativeness.

To assess determinants of anemia while accounting for the hierarchical DHS data structure, we used three-level mixed-effects logistic regression models with children nested within households and households nested within clusters [26]. Model building followed a stepwise approach: Model 0 (null) estimated the intra-cluster correlation, Model 1 included child-level variables, Model 2 added household-level variables, and Model 3 included child-, household-, and community-level variables. Survey weights were rescaled for unbiased estimates [72]. Multicollinearity was assessed using variance inflation factors (VIF < 5) [73]. The final model was selected based on the lowest AIC and BIC values and improved model fit compared to competing models [74].

Spatial analyses examined the geographic distribution of anemia. Global spatial autocorrelation was assessed using Moran’s I [21], followed by hotspot analysis (Getis-Ord Gi) to identify high- and low-prevalence clusters [20]. Kriging interpolation produced continuous prevalence surfaces, with semi variogram parameters, model selection, and cross-validation described in Supplementary Table S1 [22]. SaTScan was used to detect significant spatial clusters using a discrete Poisson model, with population at risk as the denominator [23]. Small clusters (< 20 children) were interpreted cautiously to avoid small-sample artifacts. ArcGIS was used for spatial mapping and visualization, while STATA was used for statistical and regression analyses [75, 76]. A p-value < 0.05 was considered statistically significant.

### Ethical considerations

This study used publicly available, anonymized DHS data. Ethical approval for the 2023–24 DHS was obtained from the DHS Program and the national review boards in the DRC, with informed consent obtained from caregivers [1, 2]. The datasets are publicly available at the DHS Program website: https://dhsprogram.com.

## Results

### Socio-demographic characteristics

The study included a weighted population of 11,393 children under five in the DRC. Children were fairly evenly spread across age groups, with 1,292 (11.3%) aged 0–5 months and 3,359 (29.5%) aged 6–23 months. Just over half were boys (5,975; 52.4%), and most lived in rural areas (8,077; 70.9%). Mothers’ education and household wealth varied: 5,760 children (50.6%) had mothers with secondary or higher education, and 5,203 (45.7%) came from poorer households. Most mothers were aged 25–34 years (4,878; 42.8%), and nearly half of the children lived in households of 5–7 members (5,196; 45.6%) (Table 1).

**Table 1:** Weighted Socio-Demographic Characteristics of Under-Five Children, DRC 2023/24

Characteristics	Category	Unweighted *N*	Weighted *N*	Weighted *N* (%)
Child Age (months)	0–5	1,483	1,292	11.3
	6–23	3,651	3,359	29.5
	24–35	2,441	2,174	19.1
	36–47	2,584	2,320	20.4
	48–59	2,545	2,248	19.7
Child Sex	Male	6,487	5,975	52.4
	Female	6,217	5,418	47.6
Residence	Urban	3,147	3,316	29.1
	Rural	9,557	8,077	70.9
Maternal Education	No education	3,885	2,342	20.6
	Primary	5,323	3,291	28.9
	Secondary+	3,496	5,760	50.6
Wealth Index	Poor	7,106	5,203	45.7
	Middle	2,815	2,450	21.5
	Rich	2,783	3,740	32.8
Mother’s Age (years)	15–24	3,885	3,318	29.1
	25–34	5,323	4,878	42.8
	35–49	3,496	3,197	28.1
Household Size	≤ 4 members	2,729	2,474	21.7
	≥ 5–7 members	5,786	5,196	45.6
	≥ 8 members	4,189	3,724	32.7
Characteristics	Category	Unweighted N	Weighted N	Weighted N (%)
Child Age (months)	0–5	1,483	1,292	11.3
	6–23	3,651	3,359	29.5
	24–35	2,441	2,174	19.1
	36–47	2,584	2,320	20.4
	48–59	2,545	2,248	19.7
Child Sex	Male	6,487	5,975	52.4
	Female	6,217	5,418	47.6
Residence	Urban	3,147	3,316	29.1
	Rural	9,557	8,077	70.9
Maternal Education	No education	3,885	2,342	20.6
	Primary	5,323	3,291	28.9
	Secondary+	3,496	5,760	50.6
Wealth Index	Poor	7,106	5,203	45.7
	Middle	2,815	2,450	21.5
	Rich	2,783	3,740	32.8
Mother’s Age (years)	15–24	3,885	3,318	29.1
	25–34	5,323	4,878	42.8
	35–49	3,496	3,197	28.1
Household Size	≤ 4 members	2,729	2,474	21.7
	≥ 5–7 members	5,786	5,196	45.6
	≥ 8 members	4,189	3,724	32.7

### Prevalence and severity of anemia among under-five children

The 2023–24 DHS survey in Congo, covering 12,704 children across 51 strata and 778 primary sampling units, estimated that 51.7% of under-five children were anemic (95% CI: 49.5%–53.9%), while 48.3% were not anemic (95% CI: 46.1%–50.5%). Regarding severity, mild anemia affected 25.6% of children (95% CI: 24.1%–27.2%), moderate anemia was observed in 25.0% (95% CI: 23.1%–26.9%), and severe anemia was rare, occurring in 1.1% (95% CI: 0.8%–1.5%) (Fig. 1).

### Weighted prevalence of anemia among under-five children by region and key characteristics

The highest prevalence was observed among infants aged 0–5 months (56.2%; 51.8–60.5), while children aged 24–35 months showed the lowest prevalence (50.6%; 47.4–53.7). Boys were slightly more affected than girls (52.5% vs. 50.8%), and children living in urban areas had a marginally higher burden compared to rural counterparts (53.2% vs. 51.1%).

children of mothers with no formal education and those from poorer households tended to experience higher rates of anemia. Striking differences were observed across provinces, with Haut-Lomami (64.3%), Maniema (62.8%), Tshuapa (61.4%), Bas-Uele (61.1%), and Haut-Katanga (60.9%) facing the greatest burden. In contrast, Haut-Uele (30.2%), Nord Ubangi (33.0%), and Nord-Kivu (34.0%) had the lowest prevalence (Tables 2 and Tables 3).

**Table 2:** Weighted Prevalence of Anemia Among Under-Five Children by Region and Key Characteristics, DRC DHS 2023–24

Characteristic / Region	Category	Anemic *N*	Weighted Prevalence (%) (CI)
Child Age (months)	0–5	834	56.2 (51.8–60.5)
	6–23	1,866	51.1 (4854.1)
	24–35	1,245	50.6 (47.4–53.7)
	36–47	1,358	51.0 (47.9–54.1)
	48–59	1,310	51.8 (48.1–55.5)
Sex	Male	3,411	52.5 (49.9–55.1)
	Female	3,202	50.8 (48.4–53.2)
Residence	Urban	1,612	53.2 (50.1–56.2)
	Rural	5,001	51.1 (48.3–53.9)
Maternal Education	No education	1,451	52.8 (48.7–56.9
	Primary	2,250	49.9 (46.0–54.0
	Secondary+	2,912	52.2 (49.5–54.9)
Wealth Index	Poor	3,834	53.7 (51.0–56.4)
	Middle	1,432	49.2 (44.5–53.9
	Rich	1,347	50.5 (46.7–54.3)
Province / Region	Kinshasa	204	59.0 (52.4–65.2)
	kongo central	183	57.2 (48.0–65.9)
	Kwango	248	57.6 (49.1–65.6)
	Kwango	203	45.9 (36.6–55.5)
	mai-ndombe	151	54.5 (39.3–69.0)
	Equateur	241	52.1 (43.8–60.2)
	nord Ubangi	159	33.0 (26.3–40.5)
	sud Ubangi	302	45.1 (36.6–53.9)
	Mongala	256	48.0 (39.5–56.7)
	Tshuapa	266	61.4 (57.3–65.4)
	Tshopo	237	55.0 (47.9–62.0)
	bas-uele	213	61.1 (49.5–71.5)
	haut uele	128	30.2 (15.4–50.7)
	Ituri	218	42.5 (34.5–51.0)
	nord-kivu	200	34.0 (27.3–41.4)
	sud-kivu	288	41.3 (31.8–51.4)
	Maniema	337	62.8 (54.3–70.5)
	haut-katanga	338	60.9 (51.6–69.4)
	haut Lomami	414	64.3 (55.6–72.1)
	Lualaba	365	56.4 (38.4–72.9)
	Tanganyika	259	52.2 (46.4–57.9)
	Lomami	301	53.3 (46.4–60.0)
	Sankuru	224	53.3 (45.3–61.1)
	Kasa oriental	255	53.6 (46.8–60.4)
	Kasa	295	55.3 (45.0–65.1)
	Kasa central	328	52.9 (44.8–60.7)

**Table 3:** Significant Spatial Clusters of Childhood Anemia in the DRC, 2023–24 DHS

Cluster	Location IDs (included)	Center (Lat, Lon)	Radius (km)	Population	Relative Risk	% Cases in Area	LLR	*p*-value
1 (Most likely)	174, 294, 421, 487, 430, 533, 241, 732, 558, 694, 289, 242, 268, 454, 490, 341, 182, 678, 437, 607, 618, 621, 466, 332, 104, 697, 776, 446, 80, 572, … (total > 150 IDs)	11.64°S, 27.52°E	910.16	4,579	**1.22**	58.8%	65.33	< 0.001
2	742, 247, 754, 495, 474, 222, 345, 414, 698, 329, 44, 542, 690, 428, 143, 272, 33, 492, 145, 531, 361, 644, 303, 214, 526	0.08°S, 21.41°E	183.77	443	**1.24**	63.9%	13.07	0.0015
3	488, 450, 344, 737, 113, 413, 383	4.41°S, 18.43°E	51.43	102	**1.47**	76.5%	13.02	0.0016
4	267	1.20°S, 17.16°E	0.00	19	**1.92**	100.0%	12.42	0.0027
5	166, 648, 366	7.55°S, 17.78°E	51.25	48	**1.64**	85.4%	12.01	0.004
6	463, 517, 467	5.06°S, 12.66°E	20.19	23	**1.84**	95.7%	11.00	0.01

### Spatial Autocorrelation of Childhood Anemia Prevalence

The global Moran’s I analysis demonstrated significant spatial autocorrelation in childhood anemia prevalence among under-five children in the Democratic Republic of Congo (Moran’s I = 0.095, Z = 2.84, *p* = 0.0044). This indicates that the spatial distribution of anemia was not random, with areas of high anemia prevalence tending to be located near other high-prevalence areas and areas with lower prevalence clustering together.

**Fig. 2:**
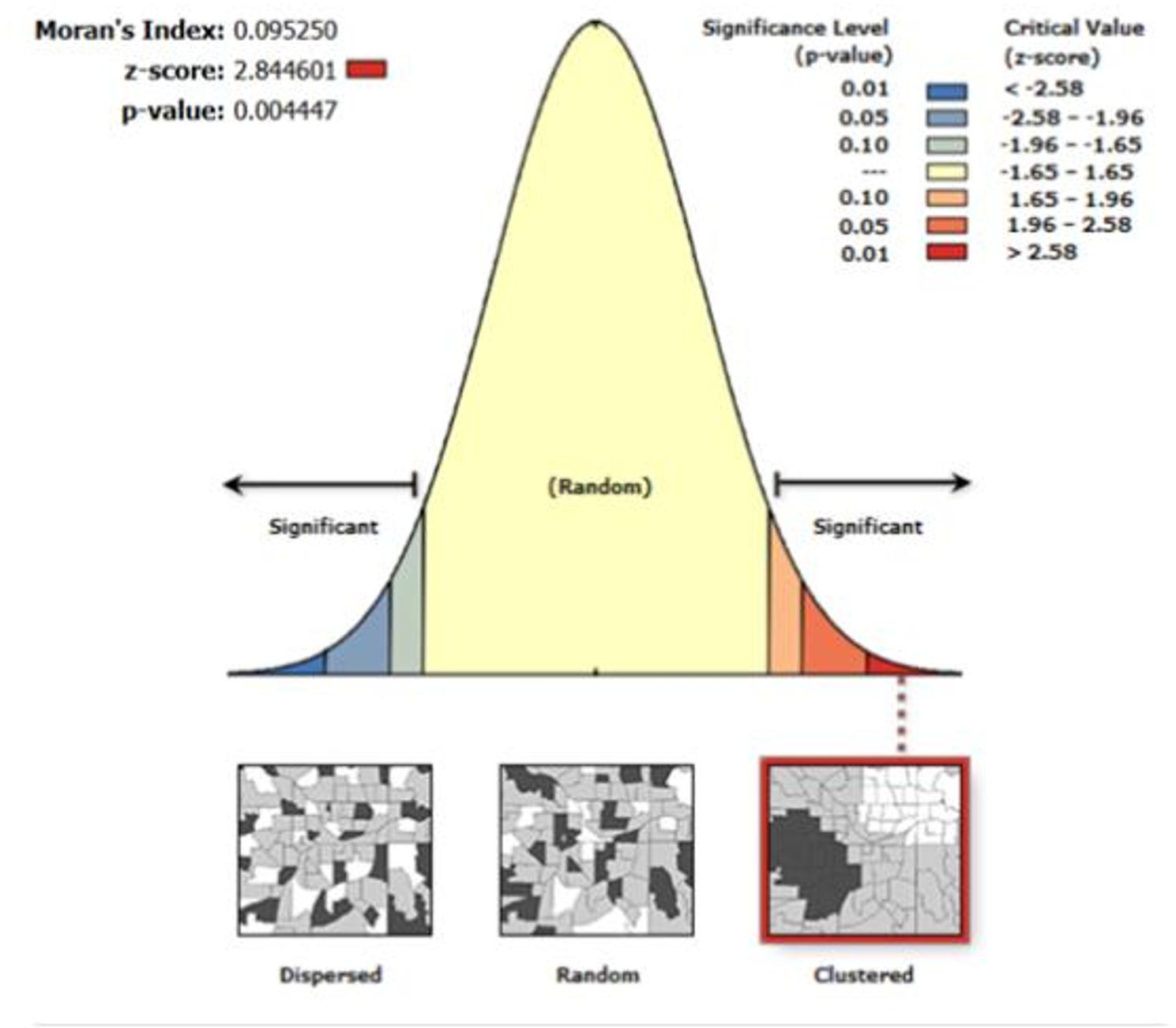
Spatial clustering of under-five anemia in the Democratic Republic of Congo

The positive Moran’s I value indicates a tendency toward spatial clustering rather than dispersion, suggesting that geographic location plays an important role in the distribution of childhood anemia. The observed z-score exceeded the critical threshold for statistical significance (Z > 2.58), confirming significant spatial clustering at the 99% confidence level (Fig. 2).

### Hotspot analysis of childhood anemia prevalence in the Democratic Republic of Congo

The Getis-Ord Gi* hotspot analysis identified significant spatial clustering of childhood anemia prevalence across the Democratic Republic of Congo. High-risk clusters (hotspots) were observed in Kinshasa and Kongo-Central, the Kasai region (Kwango, Kasai, Kasai-Central, and Kasai-Oriental), and eastern provinces including Maniema, Tanganyika, Haut-Lomami, Haut-Katanga, and South Kivu. Children residing in these geographic areas had a higher likelihood of living in communities with elevated anemia prevalence.

Conversely, significant low-risk clusters (coldspots) were identified mainly in the northern provinces, including Nord-Ubangi, Sud-Ubangi, Mongala, and Ituri, with additional localized coldspots observed in Kwilu and Kasai-Oriental. The observed spatial clustering indicates substantial geographic variation in childhood anemia burden within the country. These findings highlight the need for geographically targeted interventions focusing on high-burden areas while maintaining surveillance in regions with relatively lower anemia prevalence.

**Fig. 3:**
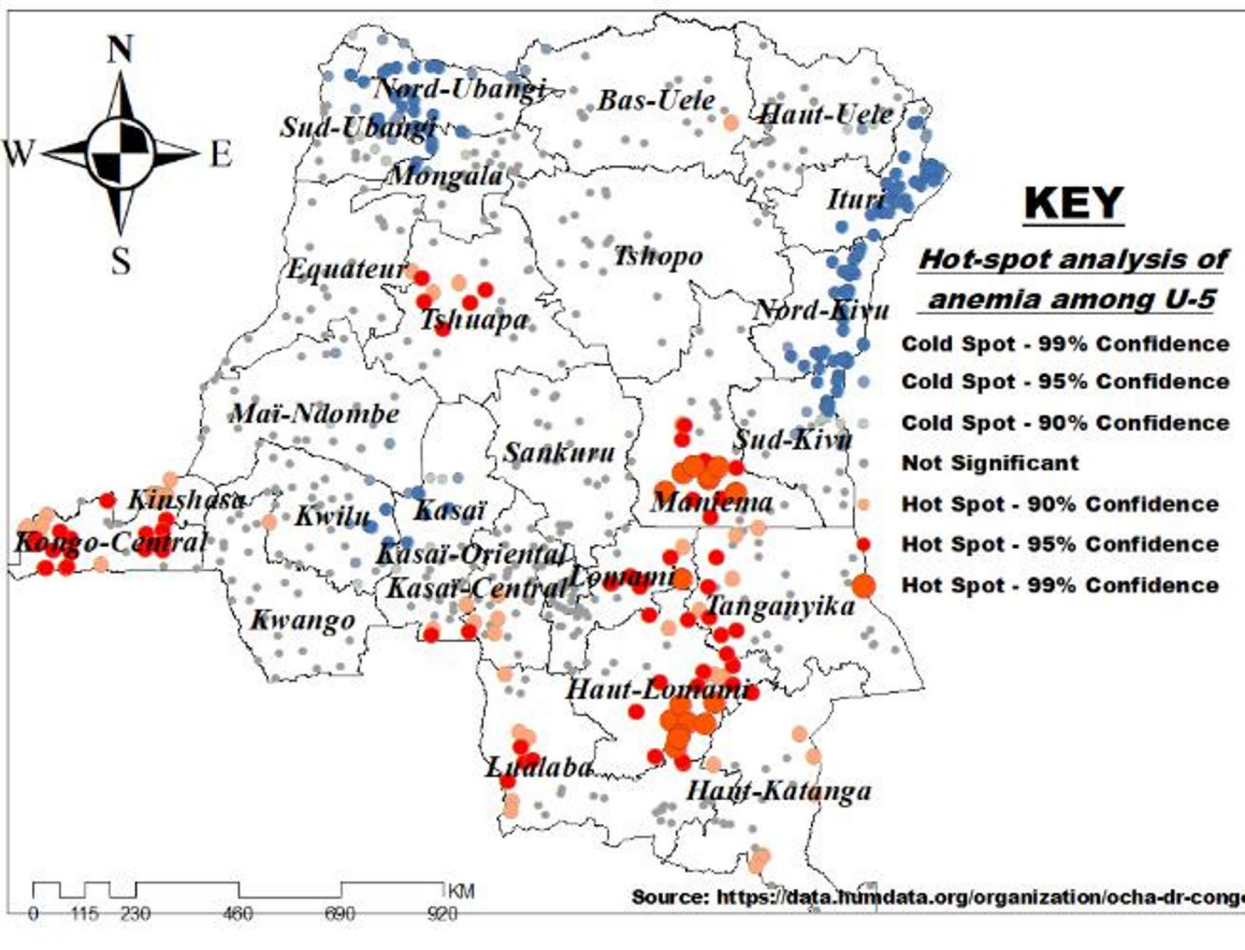
Hotspot analysis of anemia among under-five children in the DRC, 2023–2024 DHS

### Spatial prediction of childhood anemia prevalence in the Democratic Republic of Congo

The spatial prediction map demonstrated considerable geographic variation in the predicted prevalence of anemia among under-five children in the Democratic Republic of Congo. Higher predicted anemia prevalence was observed in several eastern and central provinces, including Maniema, Tanganyika, Haut-Lomami, Haut-Katanga, South Kivu, and the Kasai region, where predicted prevalence frequently exceeded 60%. Elevated predicted prevalence was also observed in Kinshasa and Kongo-Central, with estimates ranging approximately from 55% to 60%.

**Fig. 4:**
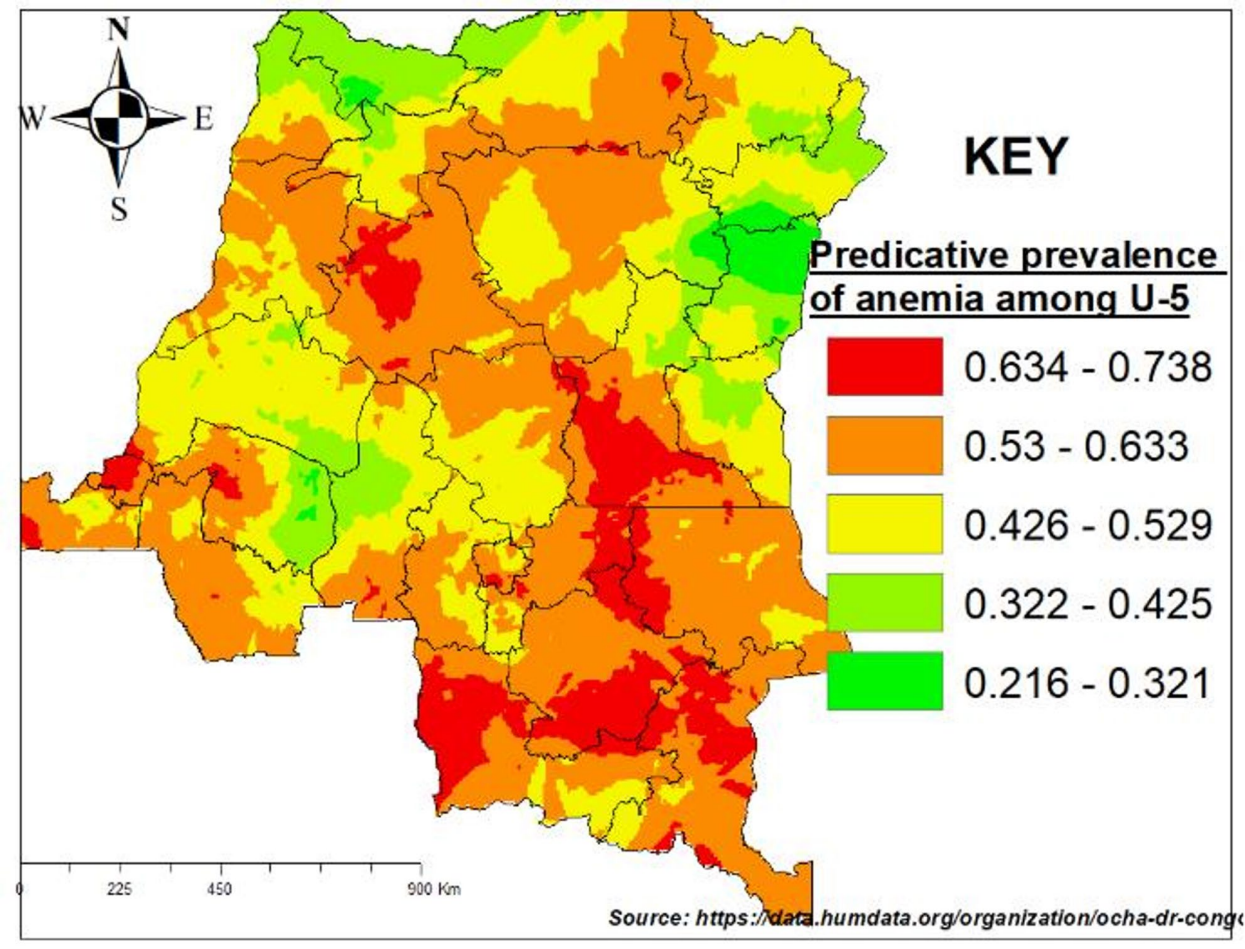
Predicted Spatial Distribution of Childhood Anemia Using Kriging Interpolation in the DRC, 2023–24 DHS

In contrast, northern provinces, including Nord-Ubangi, Sud-Ubangi, Mongala, and Ituri, showed comparatively lower predicted anemia prevalence, generally below 45%. The predicted spatial distribution suggests that childhood anemia burden is not uniformly distributed across the country but is concentrated in specific geographic areas. These findings support the prioritization of geographically targeted interventions in high-risk locations while maintaining preventive strategies and monitoring in lower-risk areas (Fig. 4).

### Spatial clustering of childhood anemia using SaTScan

The spatial analysis identified significant geographic variation in childhood anemia prevalence across the Democratic Republic of Congo. Higher-risk areas were mainly concentrated in the eastern and central regions, including Maniema, Tanganyika, Haut-Lomami, Haut-Katanga, and parts of South Kivu. Additional high-risk areas were observed in the Kasai provinces and Kwango. In contrast, lower-risk areas were identified in northern provinces, including Nord-Ubangi, Sud-Ubangi, Mongala, and Ituri, with additional localized low-risk areas in Kwilu and Kasai-Oriental.

The SaTScan spatial scan analysis detected six statistically significant clusters of childhood anemia among children aged 6–59 months, demonstrating non-random spatial clustering of anemia cases. The most likely cluster was centered at approximately 11.64°S latitude and 27.52°E longitude and included more than 150 geographic locations with 4,579 children. Children within this cluster had a significantly increased risk of anemia compared with children outside the cluster (relative risk [RR] = 1.22, *p* < 0.001), and the cluster accounted for approximately 58.8% of anemia cases.

**Fig. 5:**
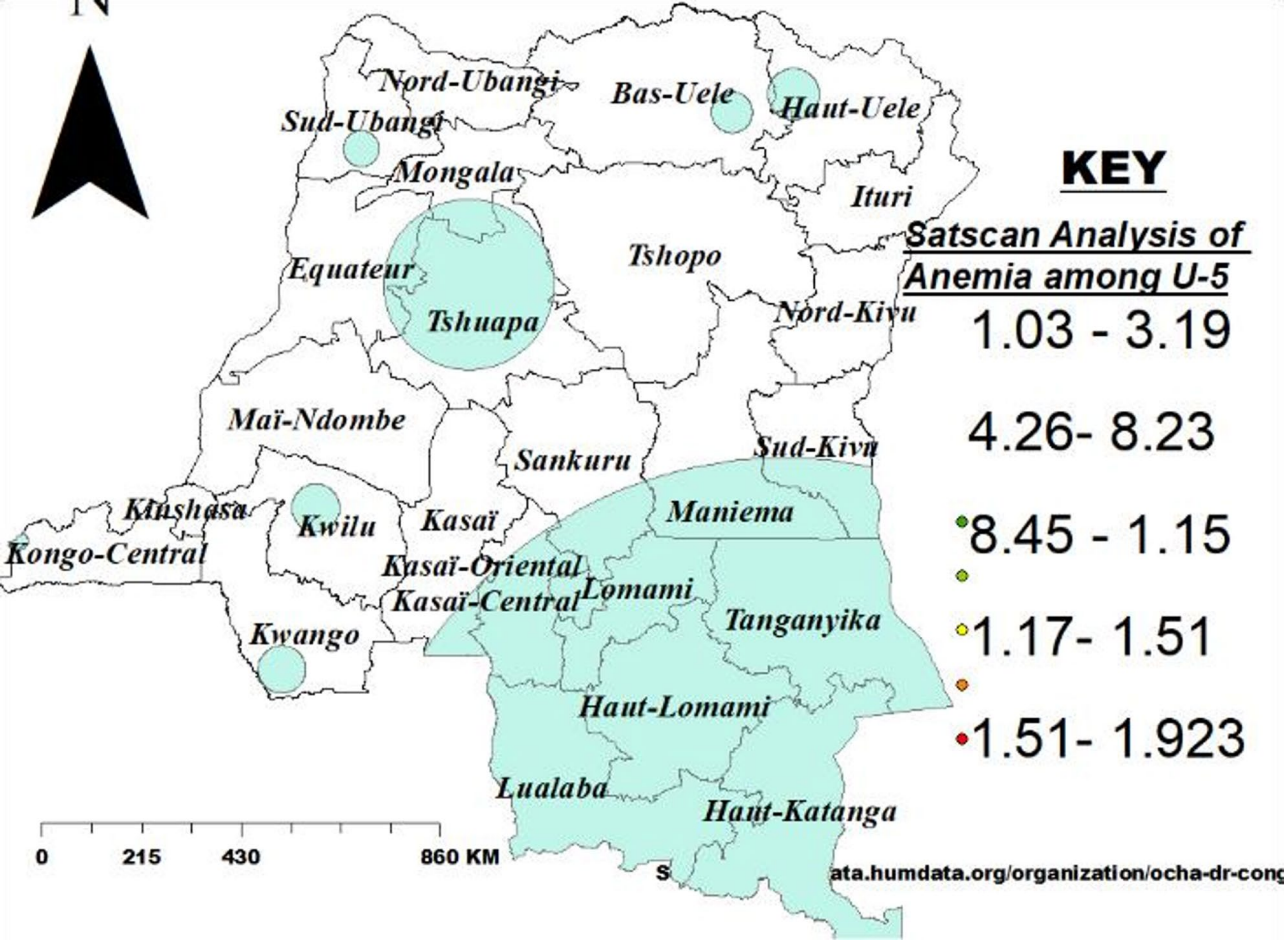
Spatial Distribution and High-Risk Clusters of Anemia Among Children Under Five in the DRC, 2023–24 DHS

Secondary clusters were also identified across different regions, with relative risks ranging from 1.24 to 1.84, indicating localized areas with substantially elevated anemia risk. One detected cluster showed an extremely high proportion of affected children, suggesting a particularly vulnerable geographic area requiring further investigation and targeted public health responses (Table 5; Fig 5).

### Determinants of childhood anemia: multilevel analysis

We examined the factors associated with childhood anemia using multilevel mixed-effects logistic regression. Children who were wasted had higher odds of anemia (Model 3: AOR = 1.29, 95% CI: 1.04–1.59*). Maternal overweight status was protective, reducing the odds of anemia (AOR = 0.66, 95% CI: 0.52–0.84*). Children from rich households were less likely to be anemic (AOR = 0.77, 95% CI: 0.59–1.00*), and households with 5–7 members were also protective (AOR = 0.84, 95% CI: 0.71–1.00*). Positive malaria status increased the odds of anemia (AOR = 1.21, 95% CI: 1.05–1.39*). Female sex (AOR = 0.89, 95% CI: 0.80–1.00+) and higher birth order (≥ 5) (AOR = 0.82, 95% CI: 0.65–1.03+) showed borderline protective effects.

Children residing in areas with high malaria risk were more likely to be anemic (AOR = 1.42, 95% CI: 1.08–1.86*), and those in low-altitude areas were more likely to be anemic (AOR = 1.39, 95% CI: 1.08–1.79*). High community poverty had a borderline protective effect (AOR = 0.72, 95% CI: 0.52–1.00+). The null model demonstrated significant clustering of childhood anemia at the community level, with an ICC of 20%, indicating that one-fifth of the total variability in anemia prevalence was attributable to differences between communities. The MOR of 2.37 suggested considerable contextual variation, meaning that a child’s risk of anemia differed substantially depending on the community of residence.

After adding individual-, household-, and community-level predictors, the ICC decreased to 11.2% and the PCV reached 44%, indicating that the included factors explained a substantial proportion of the initial community-level variation. The reduction in MOR from 2.37 to 1.85 further indicated that part of the between-community variation was explained by measured characteristics, although residual geographic and contextual differences remained (Table 4).

**Table 4:** Multilevel Mixed-Effects Logistic Regression Results for Factors Associated with Anemia among Under-Five Children

Variable	Category	Model 0		Model 1	Model 2	Model 3
Child Sex	Female	–	0.90 (0.80–1.01) +		–	0.89 (0.80–1.00) +
Wasted	Yes	–	1.29 (1.04–1.60) *		–	1.29 (1.04–1.59) *
Fever	Yes	–	1.04 (0.90–1.20)		–	1.03 (0.89–1.19)
Mother Age	25–34	–	1.00 (0.85–1.18)		–	1.00 (0.85–1.18)
	35+	–	1.05 (0.84–1.30)		–	1.05 (0.84–1.31)
Maternal BMI	Normal	–	0.94 (0.79–1.11)		–	0.94 (0.79–1.11)
	Overweight	–	0.67 (0.53–0.85) *		–	0.66 (0.52–0.84) *
Wealth	Middle	–	0.91 (0.78–1.06)		–	0.87 (0.73–1.03)
	Rich	–	0.87 (0.71–1.06)		–	0.77 (0.59–1.00) *
Household Size	5–7	–	0.84 (0.71–1.00) *		–	0.84 (0.71–1.00) *
	≥ 8	–	0.85 (0.70–1.04)		–	0.86 (0.70–1.04)
Iron-rich food	Yes	–	0.99 (0.88–1.13)		–	0.99 (0.87–1.12)
Birth Order	2nd	–	0.98 (0.81–1.19)		–	0.98 (0.81–1.19)
	3–4	–	0.91 (0.75–1.11)		–	0.92 (0.75–1.12)
	5+	–	0.81 (0.64–1.02)+		–	0.82 (0.65–1.03) +
Malaria result	Positive	–	1.30 (1.14–1.48) *		–	1.21 (1.05–1.39)*
Community Education	Medium	–	–		1.03 (0.88–1.20)	1.11 (0.90–1.38)
	High	–	–		0.93 (0.76–1.15)	0.96 (0.73–1.28)
Community Poverty	Medium	–	–		0.92 (0.74–1.13)	0.78 (0.57–1.06)
	High	–	–		0.98 (0.79–1.21)	0.72 (0.52–1.00) +
Malaria Risk	Moderate	–	–		1.04 (0.87–1.24)	0.98 (0.77–1.26)
	High	–	–		1.54 (1.27–1.86) *	1.42 (1.08–1.86) *
Altitude	Low	–	–		1.30 (1.08–1.56) *	1.39 (1.08–1.79) *
	High	–	–		0.69 (0.36–1.31)	1.43 (0.60–3.40)
Residence	Rural	–	–		0.95 (0.78–1.16)	0.85 (0.64–1.12)
Model test						
LR test	–	315.68	315.68		550.36	298.11
	ICC	20	20		13.4	11.2
	AIC	16,901	8051.2		16864.6	1088.5
	BIC	16,916	8172.		16946.5	1237.4
	PV	Ref	33		44	44
	MOR	2.37	1.98		1.85	1.85

## Discussions

Anemia remains a significant public health concern among children under five in the Democratic Republic of Congo (DRC). The 2023–24 Demographic and Health Survey (DHS) reported that 51.7% of children in this age group were anemic, with 25.6% experiencing mild anemia, 25.0% moderate anemia, and a rare occurrence of 1.1% severe anemia. The reported prevalence of anemia among under-five children in the DRC is notably high, surpassing the global average of 39.8% and the African regional average of 60% [48]. This underscores the urgent need for targeted interventions, as anemia is predominantly mild to moderate, with severe cases being rare.

The lowest prevalence was reported in Haut Uele (30.2%), Nord Ubangi (33.0%), and Nord-Kivu (34.0%), while the highest burden was seen in Haut Lomami (64.3%), Maniema (62.8%), Haut Katanga (60.9%), Tshuapa (61.4%), and Bas-Uele (61.1%). Comparable high-burden provinces in past DRC surveys and sub-Saharan Africa studies have similarly identified malaria-endemic regions and poor households as hotspots for anemia [16, 36]. These differences likely reflect variations in malaria transmission, nutritional availability, and health service access.

Age-specific prevalence was highest among infants 0–5 months (56.2%) and slightly lower but still above 50% in older children. Male children had marginally higher prevalence (52.5%) than females (50.8%), a pattern consistent with prior research showing minimal sex differences in early childhood anemia [36]. Children of mothers with no formal education (52.8%) and from poor households (53.7%) were the most affected, showing how strongly maternal knowledge and household wealth influence child nutrition. Interestingly, anemia was slightly higher in urban children (53.2%) than in rural ones (51.1%), likely reflecting dietary changes and uneven access to healthcare [53, 57].

The prevalence is consistent with previous DRC DHS surveys (> 50%), showing a continuing public health challenge. It remains high compared to the African (~ 60%) and global (~ 39.8%) averages, particularly in malaria-prone areas with limited nutrition support [34, 35, 64].

This multilevel analysis highlights that anemia among under-five children in the DRC is shaped by both individual and community-level factors. At the individual level, wasting, malaria infection, and maternal nutritional status were key predictors, while community-level malaria risk and altitude emerged as dominant determinants. These results align with sub-Saharan African evidence, including DHS-based studies and meta-analyses, showing how malnutrition and malaria interact as synergistic drivers of childhood anemia [49, 56].

Girls had slightly lower odds of anemia (AOR ≈ 0.89–0.90, borderline), echoing regional evidence that boys face higher risk due to faster growth and greater iron demands [54]. Wasted children had about 29% higher odds of anemia (AOR ≈ 1.29), aligning with evidence that acute undernutrition often coupled with infections and micronutrient deficits undermines hemoglobin production. African meta-analyses consistently link wasting with anemia risk in 6–59-month-olds [7, 60]. Maternal overweight was protective against child anemia (AOR ~ 0.66–0.67), echoing evidence that higher maternal BMI is often linked to better diet quality and energy reserves that reduce micronutrient shortfalls in children [39, 45]). Although very high BMI can increase perinatal risks and some studies suggest a U-shaped association [40] the DRC finding fits the pattern where overweight appears protective [33, 58, 65]. The observed protective association of maternal overweight with childhood anemia should be interpreted cautiously. Maternal nutritional status may partly reflect household socioeconomic conditions, improved food availability, dietary diversity, and better access to healthcare services. However, this relationship may also be influenced by residual confounding and requires further investigation.

A clear wealth gradient emerged, with showing lower odds of child anemia (AOR ~ 0.77 borderline) and middle households also trending protective [63]. This aligns with DHS-based evidence across sub-Saharan Africa linking anemia to poverty through limited diet diversity, poor living conditions, and reduced access to care [50, 54]. Malaria positivity was strongly linked with higher odds of anemia (AOR ~ 1.3), consistent with African evidence showing that hemolysis, impaired red cell production, and inflammation-driven iron sequestration all depress hemoglobin. The effect size aligns with expectations for a high-transmission context like the DRC [15, 44, 55]. Children living in high-risk malaria zones had markedly higher odds of anemia (AOR ~ 1.42). This mirrors spatial evidence showing anemia clusters aligning with malaria ecology, high vector density, and transmission intensity, which is particularly relevant in the DRC, one of the world’s highest-burden malaria settings [17, 59, 62]. Children living at low altitudes had higher odds of anemia (AOR ~ 1.30–1.39). This aligns with biological and methodological expectations: higher altitudes induce physiologic hemoglobin adaptation, and anemia definitions are adjusted for altitude, as reflected in WHO guidance [32, 38, 46, 61]. Programmatically, interventions should focus on malaria control, such as bed nets, prompt treatment, and vaccination where available, especially in high-risk, low-altitude areas. Combined with nutrition programs targeting wasting and maternal diet, these measures could meaningfully reduce childhood anemia. The association between altitude and childhood anemia may reflect differences in environmental, nutritional, and socioeconomic conditions. Lower altitude areas may experience higher malaria transmission, greater infectious disease burden, and increased exposure to environmental factors contributing to childhood anemia. Additionally, variations in food availability, dietary diversity, and health service accessibility across ecological zones may influence anemia risk.

The remaining community-level variation observed in the final model highlights the importance of context-specific and geographically targeted strategies rather than uniform approaches. Using nationally representative 2023–24 DRC DHS data, this study provides updated evidence on the magnitude, spatial distribution, and multilevel determinants of anemia among children under five. The integration of spatial analysis and multilevel modeling allowed the identification of both individual-level and community-level factors associated with childhood anemia, while also identifying geographic areas with elevated risk.

However, several limitations should be considered. The cross-sectional nature of the DHS data limits the ability to establish temporal relationships or causal associations between anemia and the identified factors. Some variables were based on caregiver-reported information and may be affected by recall or reporting bias. In addition, the limited number of children with severe anemia restricted detailed analysis of anemia severity categories. Because DHS geographic coordinates are provided at displaced cluster locations rather than exact household locations, the spatial findings should be interpreted as area-level patterns rather than precise household-level estimates.

The findings demonstrate that childhood anemia remains an important public health challenge in the DRC and is associated with both individual and contextual factors. The identified high-risk geographic clusters suggest that anemia prevention strategies may benefit from targeted approaches that integrate nutrition interventions, malaria prevention, infection control, iron supplementation, and strengthening of child health services in high-burden areas. Continued surveillance and context-specific interventions are needed to address persistent geographic inequalities in childhood anemia.

## Conclusions

Anemia remains a major public health problem among under-five children in the DRC, with substantial geographic disparities. Both individual- and community-level factors, particularly malaria exposure, wasting, household wealth, and maternal nutritional status, were associated with anemia. Targeted interventions integrating malaria control, nutritional support, and maternal health services are needed in high-burden regions.

## Data Availability

Data are publicly available via the DHS Program: https://dhsprogram.com/data/.

## Abbreviations

AIC: Akaike Information Criterion
AOR: Adjusted Odds Ratio
ArcGIS: Geographic Information System Software
BIC: Bayesian Information Criterion
BMI: Body Mass Index
CI: Confidence Interval
DHS: Demographic and Health Survey
DRC: Democratic Republic of Congo
EAs: Enumeration Areas
Gi: Getis-Ord Gi (Hotspot Statistic)
Hb: Hemoglobin
IMNCI: Integrated Management of Neonatal and Childhood Illness
LISA: Local Indicators of Spatial Association
MOR: Median Odds Ratio
SaTScan: Spatial Scan Statistics Software
STATA: Statistical Software

## References

[1] Organization WH. Global anaemia estimates. 2025.

[2] Organization WH. WHO Global Network of National Quality Control Laboratories for Pharmaceuticals: first meeting report, Rio de Janeiro, Brazil, 1–3 October 2024. World Health Organization; 2025.

[3] Komakech JJ, Nsubuga EJ, Graves JM, Apalowo OE, Mathews R, Pylate LB. Determinants of anemia among children under five in Eastern Uganda: a community-based cross-sectional study. Front Public Health. 2025;13:1618395. 40880933 10.3389/fpubh.2025.1618395 PMC12380530

[4] Tesema GA, Worku MG, Tessema ZT, Teshale AB, Alem AZ, Yeshaw Y, et al. Prevalence and determinants of severity levels of anemia among children aged 6–59 months in sub-Saharan Africa: A multilevel ordinal logistic regression analysis. PLoS ONE. 2021;16(4):e0249978. 33891603 10.1371/journal.pone.0249978 PMC8064743

[5] Kejo D, Petrucka PM, Martin H, Kimanya ME, Mosha TC. Prevalence and predictors of anemia among children under 5 years of age in Arusha District, Tanzania. Pediatric health, medicine and therapeutics. 2018:9–15. 10.2147/PHMT.S148515 PMC5804135 29443328

[6] Keokenchanh S, Kounnavong S, Midorikawa K, Ikeda W, Morita A, Kitajima T, et al. Prevalence of anemia and its associated factors among children aged 6–59 months in the Lao People’s Democratic Republic: A multilevel analysis. PLoS ONE. 2021;16(3):e0248969. 33765048 10.1371/journal.pone.0248969 PMC7993607

[7] Tadesse SE, Zerga AA, Mekonnen TC, Tadesse AW, Hussien FM, Feleke YW, et al. Burden and determinants of anemia among Under-Five children in Africa: systematic review and meta‐analysis. Anemia. 2022;2022(1):1382940. 36134386 10.1155/2022/1382940 PMC9482935

[8] Alamneh TS, Melesse AW, Gelaye KA. Determinants of anemia severity levels among children aged 6–59 months in Ethiopia: Multilevel Bayesian statistical approach. Sci Rep. 2023;13(1):4147. 36914676 10.1038/s41598-022-20381-7 PMC10011377

[9] Anteneh ZA, Van Geertruyden J-P. Spatial variations and determinants of anemia among under-five children in Ethiopia, EDHS 2005–2016. PLoS ONE. 2021;16(4):e0249412. 33793640 10.1371/journal.pone.0249412 PMC8016260

[10] Hailu BA. Mapping, trends, and factors associated with anemia among children aged under 5 y in East Africa. Nutrition. 2023;116:112202. 37832168 10.1016/j.nut.2023.112202

[11] Tekeba B, Wassie M, Mekonen EG, Tamir TT, Aemro A. Spatial distribution and determinants of anemia among under-five children in Mozambique. Sci Rep. 2025;15(1):42. 39747189 10.1038/s41598-024-83899-y PMC11696623

[12] Corsi DJ, Neuman M, Finlay JE, Subramanian S. Demographic and health surveys: a profile. Int J Epidemiol. 2012;41(6):1602–13. 23148108 10.1093/ije/dys184

[13] Merlo J, Chaix B, Yang M, Lynch J, Råstam L. A brief conceptual tutorial of multilevel analysis in social epidemiology: linking the statistical concept of clustering to the idea of contextual phenomenon. J Epidemiol Community Health. 2005;59(6):443–9. 15911637 10.1136/jech.2004.023473 PMC1757045

[14] Bahizire E, Bahwere P, Donnen P, Tugirimana PL, Balol’ebwami S, Dramaix M, et al. High Prevalence of Anemia but Low Level of Iron Deficiency in Preschool Children during a Low Transmission Period of Malaria in Rural Kivu, Democratic Republic of the Congo. Am J Trop Med Hyg. 2017;97(2):489–96. 10.4269/ajtmh.17-0030 . PubMed PMID: 28829731; PubMed Central PMCID: PMCPMC5544095. 28829731 10.4269/ajtmh.17-0030 PMC5544095

[15] Acheampong CO, Barffour MA, Schulze KJ, Chileshe J, Kalungwana N, Siamusantu W, et al. Age-specific differences in the magnitude of malaria-related anemia during low and high malaria seasons in rural Zambian children. EJHaem. 2021;2(3):349–56. PubMed PMID: 35844700; PubMed Central PMCID: PMCPMC9175671. 35844700 10.1002/jha2.243 PMC9175671

[16] Eberechukwu A. Association Between Malaria Educational Message Exposure and Malaria Prevalence Among Children in Nigeria. Walden University; 2024.

[17] Mhelembe T, Ramroop S, Habyarimana F. Determining the risk factors of malaria and anemia in children between 6 and 59 months using the joint generalized linear mixed model on the 2021 Nigeria malaria indicator survey dataset. Front Public Health. 2025;12:1503884. 39835311 10.3389/fpubh.2024.1503884 PMC11743267

[18] Anselin L. Local indicators of spatial association—LISA. Geographical Anal. 1995;27(2):93–115.

[19] Cliff AD, Ord JK. Spatial processes: models & applications. Pion London; 1981.

[20] Getis A, Ord JK. The analysis of spatial association by use of distance statistics. Geographical Anal. 1992;24(3):189–206.

[21] Moran PA. Notes on continuous stochastic phenomena. Biometrika. 1950;37(1–2):17–23. PubMed PMID: 15420245. 15420245

[22] Cressie N. Statistics for spatial data. Wiley; 2015.

[23] Kulldorff M. A spatial scan statistic. Commun Statistics-Theory methods. 1997;26(6):1481–96.

[24] Goldstein H. Multilevel statistical models. Wiley; 2011.

[25] Snijders TA, Bosker R. Multilevel analysis: An introduction to basic and advanced multilevel modeling. 2011.

[26] Geneva S, Organization WH. Haemoglobin concentrations for the diagnosis of anaemia and assessment of severity. Vitamin and mineral nutrition information system Document Reference WHO. 2011.

[27] Mairbäurl H, Kilian S, Seide S, Muckenthaler MU, Gassmann M, Benedict RK. The increase in hemoglobin concentration with altitude differs between world regions and is less in children than in adults. HemaSphere. 2023;7(4):e854. 37038466 10.1097/HS9.0000000000000854 PMC10082317

[28] Dessie G, Li J, Nghiem S, Doan T. Prevalence and Determinants of Stunting-Anemia and Wasting-Anemia Comorbidities and Micronutrient Deficiencies in Children Under 5 in the Least-Developed Countries: A Systematic Review and Meta-analysis. Nutr Rev. 2025;83(2):e178–94. 10.1093/nutrit/nuae063. Epub 2024/05/31. 38820331 10.1093/nutrit/nuae063 PMC11723162

[29] Rutstein SO, Rojas G. Guide to DHS statistics. Calverton, MD: ORC Macro. 2006;38:78.

[30] Organization WH. World health statistics 2020. 2020.

[31] Organization WH. Guideline on haemoglobin cutoffs to define anaemia in individuals and populations. World Health Organization; 2024. 38530913

[32] Accinelli RA, Leon-Abarca JA. Age and altitude of residence determine anemia prevalence in Peruvian 6 to 35 months old children. PLoS ONE. 2020;15(1):e0226846. 10.1371/journal.pone.0226846. Epub 20200115. 31940318 10.1371/journal.pone.0226846 PMC6961872

[33] Acharya SR, Timilsina D, Acharya S. Association between blood hemoglobin levels, anemia, and body mass index in children and women of Myanmar: findings from a nationally representative health study. Sci Rep. 2024;14(1):32020. 39738999 10.1038/s41598-024-83684-x PMC11685938

[34] Bain L, Awah P, Geraldine N, Kindong N, Sigal Y, Bernard N, et al. Malnutrition in sub-Saharan Africa: Burden, causes and prospects. Pan Afr med J. 2013;15:1–9. 24255726 10.11604/pamj.2013.15.120.2535 PMC3830470

[35] Baldi A, Pasricha S-R. Anaemia: worldwide prevalence and progress in reduction. Nutritional anemia: Springer; 2022. pp. 3–17.

[36] Black RE, Victora CG, Walker SP, Bhutta ZA, Christian P, de Onis M et al. Maternal and child undernutrition and overweight in low-income and middle-income countries. Lancet. 2013;382(9890):427 – 51. Epub 20130606. 10.1016/S0140-6736(13)60937-X. PubMed PMID: 23746772. 10.1016/S0140-6736(13)60937-X 23746772

[37] Burnham KP, Anderson DR. Model selection and multimodel inference: a practical information-theoretic approach. Springer; 2002.

[38] Caravedo MA, Morales ML, Tanabe M, Lopez M, White AC Jr, Cabada MM. Demographic Characteristics and Low Iron Status Markers Are Associated with Hemoglobin Levels and Anemia among Children Living at High Elevation in Cusco, Peru. Am J Trop Med Hyg. 2024;110(5):1014. 38531100 10.4269/ajtmh.23-0666 PMC11066345

[39] Chaparro CM, Suchdev PS. Anemia epidemiology, pathophysiology, and etiology in low- and middle-income countries. Ann N Y Acad Sci. 2019;1450(1):15–31. Epub 20190422. doi: 10.1111/nyas.14092. PubMed PMID: 31008520; PubMed Central PMCID: PMCPMC6697587. 31008520 10.1111/nyas.14092 PMC6697587

[40] Christian P, Smith ER. Adolescent Undernutrition: Global Burden, Physiology, and Nutritional Risks. Ann Nutr Metab. 2018;72(4):316–28. Epub 20180504. doi: 10.1159/000488865. PubMed PMID: 29730657. 29730657 10.1159/000488865

[41] Cliff AD, Ord JK. Spatial processes: models & applications. No Title); 1981.

[42] Corsi DJ, Neuman M, Finlay JE, Subramanian SV. Demographic and health surveys: a profile. Int J Epidemiol. 2012;41(6):1602–13. 10.1093/ije/dys184. Epub 20121112. 23148108 10.1093/ije/dys184

[43] Croft TN, Marshall AM, Allen CK. Guide to DHS Statistics. Rockville, Maryland, USA: ICF; 2018. DHSpr ogram com. 2018.

[44] Ehouman MA, N’Goran KE, Coulibaly G. Malaria and anemia in children under 7 years of age in the western region of Côte d’Ivoire. Front Trop Dis. 2022;3:957166.

[45] Gebreweld A, Ali N, Ali R, Fisha T. Prevalence of anemia and its associated factors among children under five years of age attending at Guguftu health center, South Wollo, Northeast Ethiopia. PLoS ONE. 2019;14(7):e0218961. 31276472 10.1371/journal.pone.0218961 PMC6611584

[46] Gonzales GF, Suarez Moreno VJ. Hemoglobin levels for determining anemia: new World Health Organization guidelines and adaptation of the national standard. SciELO Public Health; 2024. pp. 102–4. 10.17843/rpmesp.2024.412.13894 PMC11300700 39166631

[47] Hilbe JM. Logistic regression models. Chapman and hall/CRC; 2009.

[48] Kandala NIS. Anaemia in Preschool-Aged Children in DR. Congo: Finding from a Nationally Representative Survey. 2023.

[49] Kassebaum NJ, Collaborators GBDA. The Global Burden of Anemia. Hematol Oncol Clin North Am. 2016;30(2):247–308. PubMed PMID: 27040955. 27040955 10.1016/j.hoc.2015.11.002

[50] Kejo D, Mosha TC, Petrucka P, Martin H, Kimanya ME. Prevalence and predictors of undernutrition among underfive children in Arusha District. Tanzan Food Sci Nutr. 2018;6(8):2264–72. 10.1002/fsn3.798 PMC6261180 30510726

[51] Kp B. Model selection and multimodel inference. A practical information-theoretic approach. 1998.

[52] Merlo J, Chaix B, Yang M, Lynch J, Rastam L. A brief conceptual tutorial of multilevel analysis in social epidemiology: linking the statistical concept of clustering to the idea of contextual phenomenon. J Epidemiol Community Health. 2005;59(6):443–9. 10.1136/jech.2004.023473. Epub 2005/05/25. 15911637 10.1136/jech.2004.023473 PMC1757045

[53] Ministère du Plan. et Suivi de la Mise en œuvre de la Révolution de la Modernité MdlSP, International I. Democratic Republic of Congo demographic and health survey 2013–14: key findings. Demographic Health Surv. 2014:21.

[54] Moschovis PP, Wiens MO, Arlington L, Antsygina O, Hayden D, Dzik W, et al. Individual, maternal and household risk factors for anaemia among young children in sub-Saharan Africa: a cross-sectional study. BMJ open. 2018;8(5):e019654. 29764873 10.1136/bmjopen-2017-019654 PMC5961577

[55] Muriuki JM, Mentzer AJ, Mitchell R, Webb EL, Etyang AO, Kyobutungi C, et al. Malaria is a cause of iron deficiency in African children. Nat Med. 2021;27(4):653–8. 33619371 10.1038/s41591-021-01238-4 PMC7610676

[56] Mwanri L, Worsley A, Masika J. School and anaemia prevention: current reality and opportunities–a Tanzanian case study. Health Promot Int. 2001;16(4):321–31. 10.1093/heapro/16.4.321 . PubMed PMID: 11733451. 11733451 10.1093/heapro/16.4.321

[57] Organization WH, Fund UNCs. Levels and trends in child malnutrition: key findings of the 2020 edition. UNICEF/WHO/World Bank Group joint child malnutrition estimates: World Health Organization; 2020.

[58] Phillips AK, Roy SC, Lundberg R, Guilbert TW, Auger AP, Blohowiak SE, et al. Neonatal iron status is impaired by maternal obesity and excessive weight gain during pregnancy. J Perinatol. 2014;34(7):513–8. 24651737 10.1038/jp.2014.42 PMC4074453

[59] Ranjha R, Singh K, Baharia RK, Mohan M, Anvikar AR, Bharti PK. Age-specific malaria vulnerability and transmission reservoir among children. Global Pediatr. 2023;6:100085. 10.1016/j.gpeds.2023.100085 PMC10911094 38440360

[60] Sahiledengle B, Petrucka P, Desta F, Sintayehu Y, Mesfin T, Mwanri L. Childhood undernutrition mediates the relationship between open defecation with anemia among Ethiopian children: a nationally representative cross-sectional study. BMC Public Health. 2024;24(1):1484. 38831296 10.1186/s12889-024-18931-x PMC11145842

[61] Sayers DR, Witkop CT, Webber BJ. Impact of Altitude-based Hemoglobin Modification on Pediatric Iron Deficiency Anemia Screening. J Pediatr. 2020;221:196–200. 10.1016/j.jpeds.2020.02. Epub 2020/05/25. 32446480 10.1016/j.jpeds.2020.02.085

[62] Seiler J, Wetscher M, Harttgen K, Utzinger J, Umlauf N. High-resolution spatial prediction of anemia risk among children aged 6 to 59 months in low-and middle-income countries. Commun Med. 2025;5(1):57. 40038480 10.1038/s43856-025-00765-2 PMC11880423

[63] Shimanda PP, Amukugo HJ, Norström F. Socioeconomic factors associated with anemia among children aged 6–59 months in Namibia. J public health Afr. 2020;11(1):1131. 33209233 10.4081/jphia.2020.1131 PMC7649727

[64] Stevens GA, Paciorek CJ, Flores-Urrutia MC, Borghi E, Namaste S, Wirth JP, et al. National, regional, and global estimates of anaemia by severity in women and children for 2000-19: a pooled analysis of population-representative data. Lancet Glob Health. 2022;10(5):e627–39. 10.1016/S2214-109X(22)00084-5 . PubMed PMID: 35427520; PubMed Central PMCID: PMCPMC9023869. 35427520 10.1016/S2214-109X(22)00084-5 PMC9023869

[65] Yin S, Zhou Y, Li H, Cheng Z, Zhang Y, Zhang L, et al. Association of maternal BMI during early pregnancy with infant anemia: a large Chinese birth cohort. Nutr Metab (Lond). 2020;17(1):32. 10.1186/s12986-020-00448-w. Epub 20200419. 32328147 10.1186/s12986-020-00448-w PMC7169019

[66] Murphy J. Haemoglobin concentrations for the diagnosis of anaemia and assessment of severity. Vitamin and mineral nutrition information system. Geneva: World Health Organization; 2011. 2002.

[67] Organization WH. WHO child growth standards: length/height-for-age, weight-for-age, weight-for-length, weight-for-height and body mass index-for-age: methods and development. World Health Organization; 2006.

[68] Perera R, Wickremasinghe R, Newby G, Caldera A, Fernando D, Mendis K. Malaria control, elimination, and prevention as components of health security: a review. Am J Trop Med Hyg. 2022;107(4):747. 36067989 10.4269/ajtmh.22-0038 PMC9651538

[69] Filmer D, Pritchett LH. Estimating wealth effects without expenditure data—or tears: an application to educational enrollments in states of India. Demography. 2001;38(1):115–32. 11227840 10.1353/dem.2001.0003

[70] Rustein SO, Johnson K. The DHS wealth index. 2004.

[71] Macinko J, Starfield B. The utility of social capital in research on health determinants. Milbank Q. 2001;79(3):387–427. IV. doi: 10.1111/1468-0009.00213. PubMed PMID: 11565162; PubMed Central PMCID: PMCPMC2751199. 11565162 10.1111/1468-0009.00213 PMC2751199

[72] Asparouhov T. General multi-level modeling with sampling weights. Commun Statistics—Theory Methods. 2006;35(3):439–60.

[73] O’brien RM. A caution regarding rules of thumb for variance inflation factors. Qual Quant. 2007;41(5):673–90.

[74] Burnham KP. Model selection and multimodel inference. (No Title). 2004:488.

[75] StataCorp L. Stata meta-analysis reference manual. Stata: release 17 Statistical software. 2021.

[76] ESRI R. ArcGIS desktop: release 10. Volume 634. CA: Environmental Systems Research Institute; 2011. pp. 315–25.

